# *Brucella suis* carbonic anhydrases and their inhibitors: Towards alternative antibiotics?

**DOI:** 10.1080/14756366.2017.1295451

**Published:** 2017-03-08

**Authors:** Stephan Köhler, Safia Ouahrani-Bettache, Jean-Yves Winum

**Affiliations:** a Institut de Recherche en Infectiologie de Montpellier (IRIM) UMR 9004, Université de Montpellier, CNRS, Montpellier, France;; b Institut des Biomolécules Max Mousseron (IBMM) UMR 5247 CNRS, ENSCM, Université de Montpellier, Bâtiment de Recherche Max Mousseron, Ecole Nationale Supérieure de Chimie de Montpellier, Montpellier, France

**Keywords:** Metalloenzyme, anti-infective agents, carbonic anhydrase, *Brucella*, inhibitors

## Abstract

Carbonic anhydrases have started to emerge as new potential antibacterial targets for several pathogens. Two β-carbonic anhydrases, denominated bsCA I and bsCA II, have been isolated and characterized from the bacterial pathogen *Brucella suis*, the causative agent of brucellosis or Malta fever. These enzymes have been investigated in detail and a wide range of classical aromatic and heteroaromatic sulfonamides as well as carbohydrate-based compounds have been found to inhibit selectively and efficiently *Brucella suis* carbonic anhydrases. Inhibition of these metalloenzymes constitutes a novel approach for the potential development of new anti-*Brucella* agents. This review aims at discussing the recent literature on this topic.

## Introduction

*Brucella*, a non-motile Gram-negative coccobacillus, is the causative agent of brucellosis, the most widespread bacterial zoonosis, infecting livestock and humans (human incidence 500,000 cases/year). Brucellosis is endemic in countries of the Mediterranean Area, Latin America and the Middle East^1^. This pathogenic bacterium is transmitted through direct contact with infected animal tissues, inhalation of airborne bacteria and, mainly, by ingestion of contaminated and unpasteurized dairy products^2^. Due to the high degree of infectivity of the pathogenic agent, brucellosis has also been described as being the most frequently occurring laboratory-transmitted infectious disease in clinical and research laboratories.

In human brucellosis (or Malta fever), direct person-to-person spread is extremely rare and transmission may occur sexually or during breast-feeding. The disease is highly debilitating and disabling, characterized by undulant fever, night sweats, asthenia, and arthralgia. In the lack of diagnosis and appropriate antibiotics treatment, the disease may become chronic in about 50% of the cases and cause abscesses at target organs, neurological disorders, inflammation of the joints and bone destruction. Meningitis and endocarditis are often related to fatal brucellosis^3^.

Of the 12 species of *Brucella*, all of which are infecting mammals, four are pathogenic for humans: *B. melitensis*, *B. abortus*, *B. suis* and, in very few cases, *B. canis*. *Brucella* spp. are facultative intracellular, non-sporulating and non-capsulated bacteria, capable of establishing persistent infections in humans. They replicate within professional phagocytes such as macrophages and dendritic cells and are considered as stealthy pathogens, protecting themselves from the immune system of the host. Antibiotics therapy consists in general of a bi-therapy of tetracycline and rifampicin for at least 6 weeks, and much longer during the chronic stages of infection. Moreover, this microorganism has been classified as a potential bioweapon^4^. A safe and effective vaccine in humans is not available^5^, and antibiotic-resistant strains are easily conceivable and have been isolated frequently.

In the past decade, newly described species and atypical strains of *Brucella* (as *B. microti*, *B. inopinata*, *B. papionis*, and isolates from bullfrog) were isolated from unusual hosts. These strains are fast-growing, metabolically very active and more acid-resistant (AR) than the most pathogenic classical *Brucella* species, suggesting a possible advantage in survival in uncommon environments, food and hosts^6^. The pathogenic potential for humans of some of these species is yet unknown, but *B. inopinata* BO1 strain and the *B. inopinata*-like strain BO2 were isolated from human patients. Remarkably, *B. microti*, in addition to its capacity of rapid replication in human and murine macrophage-like cells, it is the first species described to be lethal in murine infections^7^. The identification of novel, more environment-associated strains and species of *Brucella* raises the question of the possible risk of infection for humans and (re-)emergence of brucellosis in countries considered at present as brucellosis-free.

On the basis of the complete genome sequences of the three main human pathogens *B*. *melitensis, B*. *suis*, and *B*. *abortus*^8–10^, and of the advancement of genomics and proteomics, the design of mechanism-based drugs by targeting defined proteins required for the growth and virulence of the pathogen has become possible^11^^,^^12^. In fact, the *Brucella* genome encodes several metalloproteins, of which at least one, histidinol dehydrogenase HDH, has been shown to be essential for intracellular bacterial growth^11^. Metalloenzymes therefore started to emerge as new potential antibacterial targets and are currently under investigation by our group^13–16^.

Among them, zinc enzyme carbonic anhydrases (EC 4.2.1.1), which are also present in many other pathogenic bacteria such as *Mycobacterium tuberculosis*^17^ and *Helicobacter pylori*^18^, have been considered as potential anti-bacterial target and started to be investigated in detail in order to identify new anti-infective agents with novel mechanisms of action^19^^,^^20^.

Among zinc metalloproteins encoded in the *Brucella* genome, two carbonic anhydrase-encoding genes (BR1829 and BRA0788 in *B. suis*) were identified and characterized, one on each of the two chromosomes^16^^,^^21–23^. These two zinc metalloenzymes of 25 kDa were shown to belong to the β-class CA family, having characteristics similar to those of other bacterial β-CAs found up to now. All conserved amino acid residues typical of β-CAs and involved in the catalytic cycle, i.e. the four zinc-binding residues, Cys42, Asp44, His98 and Cys101, are preserved. It was also demonstrated that these bacterial metalloenzymes efficiently catalyze the CO_2_ hydration to bicarbonate and protons, bsCA II being slightly more active than bsCA I (*k*_cat_/*K*_m_ of 8.9 × 10^7^ M^−1^ s^−1^ versus *k*_cat_/*K*_m_ of 3.9 × 10^7^ M^−1^ s^−1^). First studies reported also that both bsCA I and bsCA II were inhibited by many sulfonamides/sulfamates^21–23^. Following our previous review^16^, the purpose of the present manuscript is to give an overview of the latest progress in the field of *Brucella* carbonic anhydrases.

## Inhibition of bsCA I and bsCA II: an update

### Earlier studies

The first bsCA I and bsCA II inhibition studies were reported by our group and were performed on a library of the main classes of sulfonamides inhibiting human carbonic anhydrases, including 13 clinically used carbonic anhydrase inhibitors (such as acetazolamide, ethoxzolamide, dichlorophenamide, zonisamide, topiramate and sulpiride). All compounds efficiently inhibited bsCA I and also bsCA II, with inhibition constants in the nanomolar ranges (K_*I*_s of 84–923 nM)^21–23^.

In 2010, we reported the inhibitory activity of glycosylsulfanilamide against bsCA I and bsCA II, showing that both carbonic anhydrase of *Brucella suis* could be inhibited by glycoinhibitors, with inhibition constants below 10 nM for the best inhibitors. For the first time, a significant inhibition of *Brucella suis* growth was observed *in vitro* with the rhamnopyranosyl derivatives at the concentration of 100 µM as well as 10 µM in comparison with acetazolamide^24^.

In 2012, our group investigated the inhibitory activity of a wide range of inorganic anions and of various small molecule compounds known to target the zinc ion in metalloproteins against the two bacterial enzymes bsCA I and bsCA II. Both of the enzymes were inhibited in the millimolar range by the anions tested in this study, and in the low micromolar range by sulfamide, sulfamic acid, phenylboronic acid. Even if the activities observed were weak, this study allowed to have an overview on the activity of simple structures and may provide lead scaffolds for further studies and drug design^25^.

Based on these preliminary data suggesting that carbonic anhydrase inhibition may represent an alternative strategy for designing anti-*Brucella* agents, several research groups explored the possibility to develop new inhibitors based either on classical scaffold or on alternative chemotypes, in order to improve inhibitory activity, but also the selectivity against the bacterial CAs over the human enzymes.

### Carbohydrate-based *Brucella suis* CAs inhibitors

In 2015, the group of Colinas published two interesting studies describing the inhibitory capacity of a small library of *C*-cinnamoyl glycosides incorporating either a methoxy aryl or a phenol moiety against *Brucella suis* carbonic anhydrases^26^^,^^27^.

**Figure 1.**
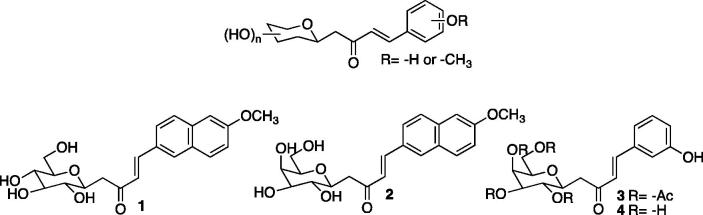
*C*-cinnamoyl glycoside inhibitors.

The inhibitory activities of the best carbohydrate-based CA inhibitors **1**, **2**, **3** and **4** (Figure 1) against human CA II and the purified bsCA I and bsCA II of the pathogen, are listed in Table 1. All compounds showed activities in the micromolar range. The most interesting feature of these two studies was the selectivity of the two most active compounds, **1** and **2**, with a preferential inhibition of bsCA I and bsCA II over human CA II.

**Table 1.** Inhibition data of compounds **1–11** against recombinant hCA II, bsCA I and bsCA II. Selectivity ratios of K*_I_* for β-CAs compared to human α-CA isozyme II.

K*_I_* (μM)	hCA II	bsCA I	bsCA II	Selectivity ratio hCA II/bsCA I	Selectivity ratio hCA II/bsCA II
**1**	>50	0.42	0.22	119	227
**2**	>50	0.43	0.21	116	238
**3**	7.1	0.68	0.63	10	11
**4**	3.1	6.54	0.83	0.5	4
**5**	>10	0.84	0.47	12	21
**6**	>10	>10	0.092	–	108
**7**	>10	>10	0.63	–	16
**8**	>10	0.95	0.75	0.5	13
**9**	0.59	0.025	0.002	24	243
**10**	0.65	0.029	0.002	22	255
**11**	0.71	0.011	0.002	62	309

**Figure 2.**
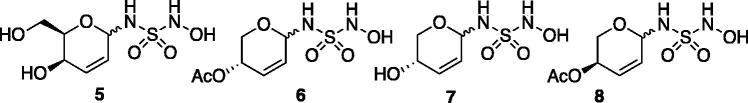
*N*-hydroxysulfamide glycoinhibitors.

The same year, our group reported glycoinhibitors in *N*-hydroxysulfamides series obtained by Ferrier sulfamidoglycosylation of glycals^28^^,^^29^. In this study, the glycoinhibitors described **5**, **6**, **7** and **8** (Figure 2) were more active on bsCA II (59.8 and 799 nM) than on the isoform bsCA I (K*_I_* ranging from 522 to 958 nM) (Table 1). Among the small library of compounds tested, some of them were shown to have an interesting selectivity profile with either (a) the preferential inhibition of the bacterial over the human CA II isoform, or (b) the inhibition of one of the bacterial isoforms over the other (Table 1).

These two studies show the importance and the opportunity to use carbohydrate scaffolds in the design of carbonic anhydrase inhibitors.

### Classical Schiff base sulfonamides

**Figure 3.**
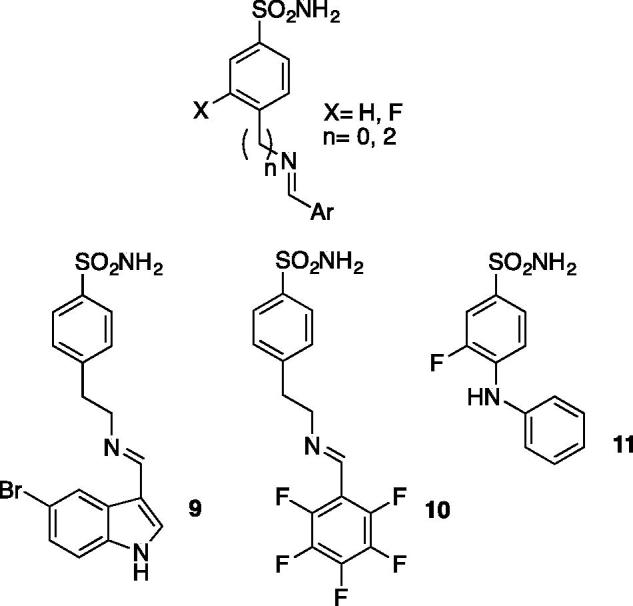
Schiff base sulfonamide inhibitors.

Recently, the group of Supuran reported activity of a series of Schiff base sulfonamides against *Brucella suis* CAs (Figure 3)^30^. Even if the structures of the inhibitors described are quite classical, very potent inhibitors were obtained with activities below 5 nM on the isoform bsCA II. The more active inhibitors **9**, **10** and **11**, were about 1–2 orders of magnitude more selective for the bacterial CA isoform (bsCA II) than for human CA II. These results confirm the ever-present interest in the exploration of classical scaffolds in sulfonamide series to identify inhibitors with higher activity and selectivity against bacterial carbonic anhydrases.

### Potential role of CA in *Brucella*: preliminary results

In a first attempt to study the physiological functions of both bsCA I (BR1829) and bsCA II (BRA0788) and/or their possible participation in virulence mechanisms of *Brucella*, our group performed separate inactivation of both bsCA-encoding genes in *B. suis* by allelic exchange between the wild-type copy of the gene and a suicide plasmid-encoded inactivated copy. In parallel, we tried to construct a double mutant by concomitant knockout of both genes, but this approach has been unsuccessful.

Using the single-gene mutants obtained in BR1829 and BRA0788 in comparison to the *B. suis* wild-type strain, we studied the possible involvement of *Brucella* CAs in growth of the bacteria (i) in rich broth, (ii) in minimal medium, and (iii) during infection of macrophage host cells. No difference of growth between wild-type and the two CA mutants could be observed under any of these experimental conditions.

Interestingly, the construction of a mutant strain of *B. suis*, where both CA-encoding genes were inactivated simultaneously, has not been possible. This indicates the essential character with at least one vital function of CA in *Brucella*, necessitating one functional copy. On the other hand, the two single CA mutants do not show phenotypes different from that of the wild-type strain, which may be explained by supposedly redundant functions of both enzymes.

Finally, several inhibitors such as compounds **3** and **4**, previously described as being active on the growth of *Mycobacterium tuberculosis*, were investigated for inhibition of growth of *Brucella suis* in rich medium and in minimal medium at the concentrations of 100 µM and 200 µM, respectively. These compounds had no inhibitory effect on the growth of the bacteria, may be due to poor membrane permeability^27^.

## Conclusions and outlook

This review gives an update on work performed on the two β-CAs of *B. suis* encoded by the genes BRA0788 and BR1829. Both CAs were shown to be catalytically active, performing CO_2_ hydration to bicarbonate and protons. These enzymes are also susceptible to selective inhibition over human CA II by a wide range of classical aromatic and heteroaromatic sulfonamides as well as carbohydrate-based compounds.

In *M. tuberculosis*, the β-CAs encoded by genes Rv3588c and Rv1284 have been shown to be required for growth *in vivo*^31^ and *in vitro*^32^, respectively. In *H. pylori*, both α- and β-CAs have been described to participate in urea and bicarbonate metabolism and in acid resistance^33^^,^^34^. As brucellae require an acidified vacuole of pH 4.0–4.5 in the early phase of host cell infection to allow initiation of intramacrophagic replication^35^, it appears conceivable that *Brucella* CAs may contribute to cytosolic pH homeostasis in the bacteria. As mentioned above, functional redundancy and the impossibility to obtain a CA double knockout mutant have not yet allowed to verify this hypothesis.

Besides their participation in fatty acids and small molecules biosynthesis, CAs also play an important role in environmental CO_2_-fixation, not only in plants but also in bacteria^36^. Remarkably, CO_2_-fixation has been reported for *Brucella abortus*, using 14CO_2_. Several strains require CO_2_ for growth, and the labeled carbon can be evidenced incorporated into amino acids and into pyrimidines^37^^,^^38^. It can be speculated that *Brucella* CAs participate in primary fixation of atmospheric CO_2_, prior to incorporation into organic molecules.

Since the human pathogen *Brucella* is reemerging in certain geographic regions and the first clinical isolates resistant to rifampicin have been described^39^, inhibition of *Brucella* CAs may constitute a novel approach for the potential development of clinically useful agents in the future.
